# When NIPT meets WES, prenatal diagnosticians face the dilemma: genetic etiological analysis of 2,328 cases of NT thickening and follow-up of pregnancy outcomes

**DOI:** 10.3389/fgene.2023.1227724

**Published:** 2023-08-02

**Authors:** Xueqi Ji, Qiongmei Li, Yiming Qi, Xingwang Wang, Hongke Ding, Jian Lu, Yan Zhang, Aihua Yin

**Affiliations:** ^1^ Guangzhou Medical University, Guangzhou, Guangdong, China; ^2^ Prenatal Diagnosis Centre, Guangdong Women and Children Hospital, Guangzhou, Guangdong, China; ^3^ Maternal and Children Metabolic-Genetic Key Laboratory, Guangdong Women and Children Hospital, Guangzhou, Guangdong, China

**Keywords:** WES, NIPT, NT thickening, CMA, prenatal diagnosis

## Abstract

**Objective:** To assess the performance of diverse prenatal diagnostic approaches for nuchal translucency (NT) thickening and to investigate the optimal prenatal screening or diagnostic action with a NT thickening of 95th percentile-3.50 mm.

**Methods:** A retrospective analysis of 2,328 pregnancies with NT ≥ 95th percentile through ultrasound-guided transabdominal chorionic villus sampling (CVS), amniocentesis, or cordocentesis obtained clinical samples (chorionic villi, amniotic fluid, and cord blood), and real-time quantitative fluorescent PCR (QF-PCR), chromosome karyotyping (CS), chromosome microarray analysis (CMA), or whole exome sequencing (WES) were provided to identify genetic etiologies.

**Results:** In this study, the incidence of chromosomal defects increased with NT thickness. When NT ≥ 6.5 mm, 71.43% were attributed to genetic abnormalities. The 994 gravidas with fetal NT thickening underwent short tandem repeat (STR), CS, and CMA. In 804 fetuses with normal karyotypes, CMA detected 16 (1.99%) extra pathogenic or likely pathogenic copy number variations (CNVs). The incremental yield of CMA was only 1.16% (3/229) and 3.37% (10/297) in the group with NT 95th percentile-2.99 mm and NT 3.0–3.49 mm, separately. Among the 525 gravidas with fetal NT thickening who underwent STR, CMA, and WES, the incremental yield of WES was 4.09% (21/513). In the group of NT 95th percentile-2.99 mm, there were no additional single-nucleotide variations (SNVs) detected in WES, while in 143 cases with NT of 3.0–3.49 mm, the incremental yield of WES was 5.59% (8/143).

**Conclusion:** In the group of NT 95th percentile-3.0 mm, since chromosomal aneuploidy and chromosomal copy number variation were the primary causes and the additional contribution of CMA and WES was not significant, we recommend NIPT-Plus for pregnant women with a NT thickening of 95th percentile-3.0 mm first. In addition, comprehensive prenatal genetic testing involving CMA and WES can benefit pregnancies with NT thickening of 3.0–3.49 mm.

## Introduction

Nuchal translucency (NT), defined as the subcutaneous accumulation of fluid behind the fetal neck, can be observed by an ultrasound scan between 10 and 13 weeks of gestation. NT measurement has been widely used as a marker of fetal abnormalities since it was first described in 1992 by Nicolaides et al. (1992). NT ≥ 95th percentile is not only associated with chromosomal abnormalities but also some genetic syndromes, as well as fetal structural defects (Taipale et al., 1997; Souka et al., 2005), such as congenital heart disease, diaphragmatic hernia, and skeletal dysplasias (Hyett et al., 1997; Bilardo et al., 1998; Snijders et al., 1998). Common aneuploidies, including trisomies 21, 18, and 13 and monosomy X, are the major chromosomal abnormalities associated with increased NT (Bilardo et al., 1998; Snijders et al., 1998; Kagan et al., 2006). The incidence of chromosomal defects increases with NT thickness from approximately 7% for those with NT between the 95th percentile for crown-rump length and 3.4 mm to 75% for NT of 8.5 mm or more (Taipale et al., 1997). Moreover, the most common genetic disorders such as 22q11 micro-deletion syndrome, Noonan syndrome, Smith–Lemli–Opitz syndrome, and congenital adrenal hyperplasia have also been reported in association with increased NT (Dane et al., 2008; Bilardo et al., 2010; Leung et al., 2011). In addition, when these abnormalities occur, there is also an increased risk of miscarriage, intrauterine fetal death, or developmental delay (Lund et al., 2015).

With the development of genetic analysis techniques, the non-invasive prenatal test (NIPT) of cell-free fetal DNA has become an option for screening chromosomal abnormalities and is available in various countries, while a diagnostic method using chorionic villus sampling (CVS) or amniocentesis still needs to be provided. Chromosomal microarray analysis can not only detect chromosome aneuploidy but also large fragment deletions or duplications and submicroscopic copy number variant (CNV) abnormalities, showing advantages over conventional karyotyping in prenatal diagnosis. Previous studies have shown different detection rates for pCNVs in euploidy fetuses with increased NT varying from 2% to 15% (Grande et al., 2015; Lund et al., 2015; Pan et al., 2016; Srebniak et al., 2016; Egloff et al., 2018; Su et al., 2019; Zhang et al., 2019; Zhao et al., 2020; Wang et al., 2022). Grande et al. (2015) concluded that, compared with karyotyping, the incremental yield of CMA was 4% (95% CI, 2%–7%) in a recent meta-analysis of 17 studies.

In recent years, prenatal exome sequencing (ES) has been shown to increase the diagnosis of single-gene diseases and improve the identification of genetic disorders in fetuses with structural abnormalities. Two large prospective studies in 2019 showed that ES provided an additional diagnosis in fetuses with isolated increased NT (≥3.5 mm) with diagnostic rates of only 3.2% and 3.0%, respectively (Lord et al., 2019; Petrovski et al., 2019). Other studies (Choy et al., 2019; Sparks et al., 2020; Xue et al., 2020; Yang et al., 2020; Mellis et al., 2022) [24–27] have reported the diagnostic rate in fetuses with isolated increased NT ranging from 1% to 17%, which could not be detected by CMA.

However, there is currently no global consensus on the cutoff value for when we recommend that patients choose to do CMA only or both CMA and WES when undergoing prenatal diagnosis in our daily genetic counseling.

In this study, we retrospectively analyzed the prenatal diagnosis results of NT-thickened fetuses diagnosed in our center, including CS, CMA, and WES results, and explore feasible methodological test options for each NT thickening range (especially in the controversial range of NT 95th percentile-3.0 mm and 3.0–3.49 mm) based on the analysis of different detection methods and pregnancy outcome in different NT thickening ranges.

## Materials and methods

### Demographics data

Clinical indications included 2,328 pregnant women with fetuses with increased NT in the Guangdong Women and Children Hospital between January 2019 and January 2023 for invasive prenatal diagnosis. Those women with fetal increased NT were transferred to a large tertiary referral center, Guangdong Women and Children Hospital of Guangdong Province, through a referral network in Guangzhou, China. Through ultrasound-guided transabdominal chorionic villus sampling (CVS), amniocentesis, or cordocentesis clinical samples (chorionic villi, amniotic fluid, and cord blood) were obtained. STR, G-banded karyotyping, CMA, and WES were provided to identify the fetal anomalies. Each participant signed a written informed consent form after accepting detailed pretest genetic counseling. This study was approved by the Ethics Committee of Guangdong Women and Children’s Hospital.

### QF-PCR

We performed QF-PCR to rapidly analyze T21, T18, T13, and sex chromosome aneuploidy. QF-PCR for detecting common chromosome numerical anomalies was conducted using a modified version of the previous report (Liu et al., 2019). Gel electrophoresis was performed using an ABI3100 with a 3100-POP4 gel of internal lane standard 600 as the molecular standard. The 22 polymorphic sites of chromosomes 21, 18, 13, X, and Y were selected from the NCBI database as primer sequences, and then the 5′end of the forward primer of the detection site was labeled with different fluorescence. The primer labeling and synthesis were completed by Dalian Baobio.

### G-banding karyotyping

We performed standard G-banding karyotyping to analyze the structures of all the chromosomes in the fetuses. G-banding karyotyping was conducted as in previous literature by Hu et al. (2022). Cells were cultured and prepared for G-banding and fluorescence in situ hybridization (FISH) following standard protocols. Karyotypes were described based on the criteria of the International System for Human Cytogenetic Nomenclature (ISCN 2020) (Liehr, 2021). When suspected low-level mosaicism was observed, an interphase FISH of uncultured amniocyte cells was recommended to be performed.

### Chromosomal microarray analysis

We performed chromosome microarray analysis to detect CNVs in the fetuses. Fetal uncultured genomic DNA was extracted using a DNA extraction kit (QIAamp DNA Mini Kit, QIAGEN, Germany). CMA was conducted as in previous literature (Huang et al., 2021; Hu et al., 2022), which was performed using a whole-genome CytoScan 750K array (Thermo Fisher Scientific, United States). The raw data analyzed with the Chromosome Analysis Suite 4.0 (Thermo Fisher Scientific, United States) were checked and compared carefully with the genome version GRCh37/hg19. According to the deletion and duplication in chromosome location, the clinical significances of chromosomal abnormalities were evaluated and defined as five types of properties, including pathogenicity, likely pathogenicity, benign, likely benign, and variant of uncertain significance.

### Whole exon sequencing

Fetal uncultured genomic DNA was extracted from chorionic villus samples, amniotic fluid, tissue samples, or cord blood, using the QIAamp DNA Blood MiniKit (Qiagen Sciences, United States) according to the manufacturer’s instructions. Whole-exon sequencing and Sanger sequencing (NextSeq 2000, Illumina, United States) were performed on DNA samples. Variants were identified by sequence alignment with the NCBI Reference Sequence; the pathogenicity of the identified variant was assessed based on the adapted American College of Medical Genetics and Genomics (ACMG) guidelines (Chen et al., 2022; Qian et al., 2022).

### Statistical analysis

Statistical analysis was performed using SPSS 19.0 (Chicago, United States). Categorical variables were presented as percentages.

## Results

In total, 2,328 pregnancies with a NT ≥ 95th percentile were analyzed. The mean gestational week of prenatal diagnosis was 15.5 weeks (range 11–34 weeks) with a median NT of 3.3 mm (range 2.3 mm–13.5 mm). The number of pregnant women who underwent amniocentesis or umbilical vascular puncture after 16 weeks was 1,285 cases. In 1,043 of the cases, the testing was performed by chorionic villous sampling at 11–13 + 6 weeks. The 99th percentile was defined as NT ≥ 3.5 mm for all gestational ages.

### Distribution of NT thickness

**FIGURE 1:**
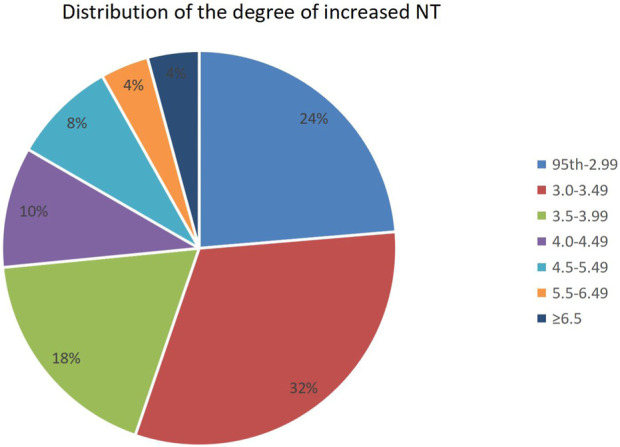
Distribution of the degree of increased NT. NT, nuchal translucency

Of all fetuses with NT ≥ 95th percentile found in early pregnancy, 551 (23.7%) had a NT between the 95th percentile and 1.99 mm, 735 (31.6%) had an increased NT of 3.0–3.49 mm, and 1,007 (53%) had a NT ≥ 95th percentile. The distribution of different NT thicknesses is shown in Figure 1.

### Genetic abnormalities

**FIGURE 2:**
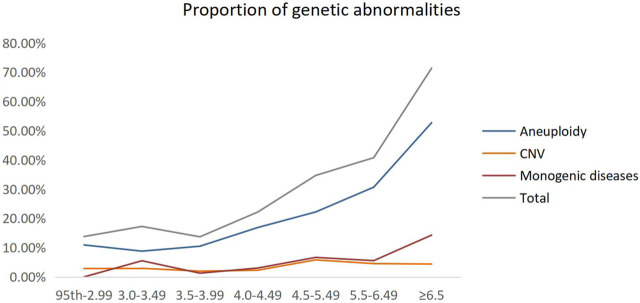
Proportion of genetic abnormalities for different NT thicknesses. CNV, copy number variant.

We grouped these fetuses with increased NT according to different NT thicknesses and calculated the percentage of genetic factors at different NT thicknesses separately. The proportion of genetic abnormalities increased with increasing NT thickness, while 13.79% was caused by genetic abnormalities, when NT increased 95th percentile-2.99 mm. When NT increased by ≥ 6.5 mm, the genetic abnormalities accounted for 71.43% (Table 1; Figure 2).

**TABLE 1:** Genetic abnormalities associated with fetal increased NT.

NT (mm)	Genetic abnormalities			Total (%)
	Aneuploidies	pCNVs	Monogenic diseases	
95th-2.99	57/522 (10.92)	15/522 (2.87)	0/52 (0.00)	13.79
3.0–3.49	63/716 (8.80)	21/716 (2.93)	9/163 (5.52)	17.25
3.5–3.99	43/410 (10.49)	8/410 (1.95)	2/158 (1.27)	13.70
4.0–4.49	37/219 (16.89)	5/219 (2.28)	3/99 (3.03)	22.21
4.5–5.49	42/189 (22.22)	11/189 (5.82)	5/75 (6.67)	34.71
5.5–6.49	27/88 (30.68)	4/88 (4.55)	2/36 (5.56)	40.78
≥6.5	48/91 (52.75)	4/91 (4.40)	3/21 (14.29)	71.43

Data are given as n (%) or %; NT, nuchal translucency; pCNVs, pathogenic copy number variants.

### Incremental yield of different genetic techniques

In this study, 994 pregnant women with fetal increased NT chose to perform STR, CS, and CMA simultaneously at the time of prenatal diagnosis, including 332 pregnant women with NT ≥ 3.5 mm. In 248 cases with euploidy fetuses, CMA detected additional pCNVs in three cases, with an increment of 1.21% (3/248) compared with CS. Among the 314 fetuses with increased NT of 95th percentile-2.99 mm, 259 were euploidy fetuses, and the additional detection rate of pCNVs by CMA versus CS was only 1.16% (3/259). Of the 297 euploidy fetuses with increased NT of 3.0–3.49 mm, the incremental yield of pCNVs by CMA was only 3.37% (10/297) (Table 2).

**TABLE 2:** Copy number variants (CNVs) detection rates in 804 euploid fetuses with increased NT.

NT (mm)	Fetuses with normal karyotype n	All CNVs detected by CMA n (%)	P/LP CNVs n (%)	VOUS n (%)	Others n (%)
95th-2.99	259	13 (5.02%)	3 (1.16%)	9 (3.47%)	1 (0.39%)
3.0–3.49	297	19 (6.4%)	10 (3.37%)	6 (2.02%)	3 (1.01%)
≥3.5	248	12 (4.83%)	3 (1.21%)	4 (1.61%)	5 (2.02%)
Total	804	44 (5.47%)	16 (1.99%)	19 (2.36%)	9 (1.12%)

Data are given as n (%) or n; NT, nuchal translucency; CMA, chromosomal microarray analysis; P/LP CNVs, pathogenic/likely pathogenic copy number variants; VOUS, variant of uncertain significance.

In our study, there were 525 fetuses with increased NT who underwent STR, CMA, and WES simultaneously. In 335 fetuses with NT ≥ 3.5 mm, there were 325 euploidy fetuses with normal CMA, and the additional detection increment of pathogenic/likely pathogenic variation (P/LP) of WES concerning CMA was 4% (13/325). Among the 45 fetuses with a NT 95th percentile-2.99 mm, all of which were euploidy fetuses with normal CMA, no additional pathogenic or likely pathogenic variations were detected in WES. Among the 145 fetuses with NT values between 3.0 and 3.49 mm, 143 were euploidy fetuses with normal CMA and eight pathogenic or likely pathogenic variations (P/LP) were detected in WES, which CMA could not identify. The additional increment yield was 5.59% (8/143) (Table 3). In addition, 53 pathogenic or potentially pathogenic variants unrelated to the fetal phenotype were unexpectedly identified in WES, with an additional incremental yield of 14.42% (74/513) compared with CMA.

**TABLE 3:** The incremental yield of whole exome sequencing (WES) in 513 fetuses with increased NT.

NT (mm)	Fetuses with normal CMA n	All variants detected by WES n (%)	P/LP variants n (%)	VOUS n (%)
95th-2.99	45	1 (2.22%)	00)	1 (2.22%)
3.0–3.49	143	15 (10.49%)	8 (5.59%)	7 (4.90%)
≥3.5	325	24 (7.38%)	13 (4.00%)	11 (3.38%)
Total	513	40 (7.80%)	21 (4.09%)	19 (3.70%)

Data are given as n (%) or n; NT, nuchal translucency; WES, whole exome sequencing; CMA, chromosomal microarray analysis; P/LP, pathogenic/likely pathogenic; VOUS, variant of uncertain significance.

### Pregnancy outcomes

We followed up on the pregnancy outcomes of 511 pregnant women with fetal increased NT who underwent concurrent STR or CS, CMA, and WES at the time of prenatal diagnosis, all of whom had negative results. Table 4 shows the pregnancy outcomes of fetuses with different degrees of increased NT for which no genetic factors have been found. Among 511 fetuses, the number of pregnant women who chose to terminate their pregnancy increased with increased NT thickness. 92.76% (474/511) had positive pregnancy outcomes (no special conditions) and adverse pregnancy outcomes (miscarriage/intrauterine death) occurred in fewer than 1%. The probability of positive pregnancy outcomes in pregnant women with different degrees of increased NT thickness did not differ between groups and was statistically significant (Table 4).

**TABLE 4:** Pregnancy outcomes in 511 fetuses with increased NT without genetic abnormalities.

NT (mm)	No special conditions	TOP	IUFD	MC	Missed
95th-2.99	45/49 (91.80)	0 (0.00)	0 (0.00)	0 (0.00)	4/49 (8.20)
3.0–3.49	133/140 (95.00)	2/140 (1.43)	2/140 (1.43)	0 (0.00)	3/140 (2.14)
3.5–3.99	126/135 (93.33)	4/135 (2.96)	1/135 (0.74)	0 (0.00)	4/135 (2.96)
4.0–4.49	78/82 (95.12)	1/82 (1.22)	0 (0.00)	0 (0.00)	3/82 (3.66)
4.5–5.49	56/62 (90.32)	4/62 (6.45)	0 (0.00)	1/62 (1.61)	1/62 (1.61)
5.5–6.49	24/27 (88.89)	2/27 (7.41)	1/27 (3.70)	0 (0.00)	0 (0.00)
≥6.5	12/16 (75.00)	4/16 (25.00)	0 (0.00)	0 (0.00)	0 (0.00)
Total	474/511 (92.76)	17/511 (3.33)	4/511 (0.78)	1/511 (0.19)	15/511 (2.94)

Data are given as n (%); NT, nuchal translucency; TOP, termination of pregnancy; IUFD, intrauterine fetal death; MC, miscarriage.

## Discussion

NT measurement is one of the common screening aids for aneuploidy, and recent studies have shown that NT thickening is associated not only with chromosomal aneuploidy but also CNVs, structural abnormalities, and some monogenic disorders. Most of the abnormalities in those with thickened NT are chromosomal aneuploidy and CNVs. Therefore, invasive prenatal diagnosis is recommended for fetuses with increased NT, mainly to detect karyotypes and CNVs.

Previous studies have shown (Bardi et al., 2020) that the proportion of genetic causes of NT thickening is correlated positively with increasing NT thickness. When increased NT of the 95th percentile was 3.5 mm, the percentage of genetic abnormalities was about 15.3%. When increased NT of 3.5–6.4 mm and ≥6.5 mm, the genetic abnormalities were 42.1% and more than 65.6%, respectively. In our study, we demonstrated that the proportion of increased NT caused by genetic abnormalities increased with increasing NT thickness, up to 71.43% at a NT ≥ 6.5 mm, of which chromosomal aneuploidy abnormalities accounted for the majority. Interestingly, we found that the difference in pCNVs in groups of NT 95th percentile-2.99 mm, 3.0–3.49 mm, 3.5–3.99 mm, and 4.0–4.49 mm was not significant. However, the percentage of pCNVs and monogenic disease were elevated when NT ≥ 4.5 mm, showing the necessity for combining CMA and WES for NT ≥ 4.5 mm. Currently, different countries have different criteria for the cutoff value of increased NT (Su et al., 2021), most countries use NT ≥ 3.5 mm or the 99th percentile as the cutoff value of increased NT, while some countries use the 95th percentile. Most scholars in China use NT ≥ 3.0 mm or ≥3.5 mm as the standard. The American College of Obstetricians and Gynecologists (ACOG) and the Society for Maternal-Fetal Medicine (SMFM) currently recommend 3.0 mm or the 99th percentile as the cutoff value and recommend detailed genetic counseling for these pregnancies, either by NIPT or invasive prenatal diagnosis (American College of Obstetricians and Gynecologists’ Committee on Practice Bulletins—Obstetrics, 2020; Prabhu et al., 2021). While NT ≥ 3.5 mm is an internationally recognized sign for invasive testing, there is no consistent standard on genetic counseling for pregnant women with NT < 3.5 mm, and research literature is scarce on prenatal screening or diagnosis for a NT 95th percentile-3.5 mm.

In the present study, we found that in the NT 95th percentile-2.99 mm group, aneuploidy and pCNVs accounted for 10.92% and 2.87%, respectively, while the percentage of monogenic disease detected by WES was 0%, which may be related to the low number of fetal cases in this group. Moreover, in 259 fetuses with a NT 95th percentile-2.99 mm, CMA detected 13 CNVs that were not detectable by conventional karyotyping, including only three pCNVs (1.16%). In the previous studies, Wang et al. (2022) found that chromosomal aneuploidy and genomic imbalance were the primary fetal abnormalities when NT was 2.5–3.0 mm and that for fetuses with increased isolated NT between 2.5 and 3.0 mm, NIPT (3.98%) had a similar rate of karyotype detection (5%) in a study of 201 fetuses with examined by NIPT and karyotype. Xie et al. (2022) applied NIPT-Plus to 72 fetuses with increased NT and achieved a sensitivity and specificity of 95.2% and 100% for common chromosomal aneuploidy, respectively. In addition, only two out of five pCNVs cases could be detectable with NIPT-Plus, which may be related to the detection platform and the different definitions. Although in our study we did not have statistics on the missed diagnosis of NIPT applied to increased NT fetuses, NIPT techniques based on high-throughput sequencing are now widely used to screen for fetal chromosomal aneuploidy and a previous study (Taylor-Phillips et al., 2016) showed that the sensitivity of NIPT was 99.3%, 97.4%, and 97.4% for trisomy 21, trisomy 18 and trisomy 13, respectively. NIPT-Plus has a combined sensitivity of 99.2% for the detection of common chromosomal microduplications and microdeletions, and a high composite positive predictive value of 92.9% for Digeorge syndrome (Liang et al., 2019). The detection rates of aneuploidy and CNVs in fetuses with NT 95th percentile-2.99 mm were high, while the incremental yield of monogenic disorders was 0%. Therefore, we suggest, as most of the literature (Wang et al., 2022; Xie et al., 2022; Yin et al., 2022) recommends that instead of invasive prenatal diagnosis for those fetuses, NIPT-Plus, which not only has high sensitivity and specificity for aneuploidy and CNVs, but also has already in large-scale clinical use in China and abroad, may be performed first especially if other structural abnormalities have not been added at the time of NT measurement. If the non-invasive results are negative, the invasive prenatal diagnosis can be left alone and follow-up ultrasound monitoring, including systemic ultrasound test and cardiac ultrasound test, can be performed to assess pregnancy outcome.

To assess the risk of chromosomal abnormalities in fetuses with NT of 3.0–3.49 mm and to determine whether invasive prenatal testing is necessary, our results showed an 11.7% risk of chromosomal abnormalities (including aneuploidy and pCNVs). The study by Petersen et al. (2020) performed a meta-analysis of literature cases with NT of 3.0–3.49 mm and a retrospective study of two groups of pregnant women with invasive testing and CMA, which showed 13.5% of 522 fetuses were diagnosed with chromosomal aberrations, similar to the results of our study. In contrast, in the study by Zhang et al. (2019), only approximately 7% were diagnosed. Combined with the conclusion of a previous study (Sagi-Dain et al., 2021) that NIPT in isolated NT of 3.0–3.49 mm would have a 1.9% missed diagnosis rate, the meta-analysis by Petersen et al. (2020) reported a 2.8% miss rate for five-chromosome NIPT and a 0.2% miss rate for NIPT, and the fact that these low rates did not distinguish isolated or non-isolated NT thickening, it is reasonable to assume that invasive prenatal diagnosis in such fetuses is necessary. Furthermore, in this study, we found that all chromosomal abnormalities detected by karyotype were detectable by CMA, with the additional detection of total CNV increment and extra detection of pCNVs increment of 6.40% (19/297) and 3.37% (10/299), indicating the additional diagnostic value of CMA in NT of 3.0–3.49 mm. Therefore, it may be possible the karyotyping can be replaced by the CMA technique which could detect both chromosomal aneuploidy and large deletion repeats and submicroscopic copy number variants (CNV). In addition, we performed WES in 143 fetuses with NT of 3.0–3.49 mm with normal CMA, and eight clinically significant pathogenic or likely pathogenic variants were identified, showing an incremental yield of 5.59%, which has not been reported previously in this range. The associated monogenic diseases have been presented in Supplementary Table S2. It is necessary to use prenatal ES for the fetus with NT of 3.0–3.49 mm. As stated by Petersen et al. (2020) and Maya et al. (2017), we recommend a threshold of 3.0 mm rather than 3.5 mm for invasive testing, and for NT of 3.0–3.49 mm comprehensive genetic testing, including CMA and WES, testing is provided to avoid a missed diagnosis and provide earlier diagnosis of clinically important chromosomal aberrations.

Furthermore, we unexpectedly found 53 additional cases of pathogenic variants irrelevant to the fetal phenotype in WES versus CMA for cases with NT thickening ≥95th percentile. In total, the additional incremental yield of WES versus CMA was 14.42% (74/513). In this regard, we can be informed that WES detects many monogenic syndromes associated with increased NT. But since NT thickening is not a disease-specific phenotype (no relevant hotspot disease), it seems less work to use a single panel genetic testing technique for NT-thickened fetuses, while using WES may reveal many other variants that are not associated with the fetal phenotype and also contain variants with pathogenicity uncertainty, and this uncertainty may increase maternal anxiety and still require follow-up ultrasound to provide accurate interpretation of WES results of unknown significance. As mentioned by Yang and Li (2022), this is a limitation of the WES application. The prenatal phenotype may be different from the postnatal phenotype, and interpreting the variants of unknown pathogenicity significance (VUS) may be complex. Whether reporting VUS is more beneficial than bad for the patient or more harmful than good is also something we need to consider carefully in our clinical work. Data from larger samples of studies are still needed to elucidate further, but we believe that as the application of WES develops and the database is constantly updated and improved, the number of variants of unknown significance will decrease.

In clinical practice, another question we always face is how to perform genetic counseling when a pregnant woman with fetal increased NT has chosen to undergo an interventional prenatal diagnosis and has undergone comprehensive genetic testing with negative results (i.e., no relevant cause of the thickening is found or it is considered tentatively to be due to a non-genetic abnormality). Based on this, we followed up 511 fetuses with NT thickening that underwent concurrent STR or CS, CMA, and WES, all with negative pregnancy outcomes. Notably, regardless of NT thickness and without differentiating isolated or non-isolated NT thickening, more than 90% of fetuses with NT thickening without genetic cause had a good pregnancy outcome, especially when NT thickening was less than 6.5 mm. It is generally consistent with what has been reported in other literature (Bilardo et al., 2007). When NT ≥ 6.5 mm, most fetuses may be diagnosed with lymphoedema cysticercosis, which has a poor pregnancy outcome (Graesslin et al., 2007). We speculate that this may account for the decreasing proportion of good pregnancy outcomes.

## Conclusion

In the group of NT 95th percentile-3.0 mm, since chromosomal aneuploidy and chromosomal copy number variation are the primary causes and the additional contribution of CMA and WES is not significant, we recommend NIPT-Plus for pregnant women with NT thickening of 95th percentile-3.0 mm first. In addition, comprehensive prenatal genetic testing involving CMA and WES can benefit pregnancies with NT thickening of 3.0–3.49 mm.

## Data Availability

The original contributions presented in the study are included in the article/Supplementary Material, further inquiries can be directed to the corresponding author.
