# Supplementary material for: When NIPT meets WES, prenatal diagnosticians face the dilemma: genetic etiological analysis of 2,328 cases of NT thickening and follow-up of pregnancy outcomes
Source: Front Genet. 2023 Aug 2;14:1227724. doi: 10.3389/fgene.2023.1227724 (PMC10433188; doi:10.3389/fgene.2023.1227724)
Supplement: Supplementary file 1 [file DataSheet1.docx]

Supplementary Material

# Supplementary Data

Supplementary Material should be uploaded separately on submission. Please include any supplementary data, figures and/or tables.

Supplementary material is not typeset so please ensure that all information is clearly presented, the appropriate caption is included in the file and not in the manuscript, and that the style conforms to the rest of the article.

# Supplementary Figures and Tables

## Supplementary Tables

Table S1. Details of copy number variant identified by CMA in 44 euploid fetuses with increased NT

| **Case ID** | **NT（mm)** | **QF-PCR result** | **Karoytype result** | **CMA result** | **Size** | **Related syndrome/gene** | **Inheritance** | **Categorization** | **outcome** |
| --- | --- | --- | --- | --- | --- | --- | --- | --- | --- |
| 1 | 3.10 | Nomal | Nomal | arr[hg19] 8q24.13q24.21(127,087,342-128,404,823)x3 | 1.3Mb | No specified | N/S | VOUS | Live birth |
| 2 | 3.00 | Nomal | Nomal | arr[hg19] 16p13.11(15,375,911-16,278,133)x3 | 902kb | 6 OMIM genes | maternal | VOUS | Live birth |
| 3 | 3.10 | Nomal | Nomal | arr[hg19] 7q11.21q11.23(62,569,501-75,233,244)×2 hmz | 12.7Mb | AOH | De novo | Others-AOH | Live birth |
| 4 | 3.60 | Nomal | Nomal | arr[hg19] 22q11.21(18,648,855-20,312,661)x3 | 1.7Mb | 22q11.2 microduplication syndrome | De novo | P | Live birth |
| 5 | 3.30 | Nomal | Nomal | arr[hg19] Xq22.2(102,957,372-103,111,680)x2 | 154kb | 2 OMIM genes （RAB9B , PLP1）；Pelizaeus-Merzbacher disease（PMD）、Spastic paraplegia 2, X-linked（SPG2） | N/S | P | TOP |
| 6 | 3.20 | Nomal | Nomal | arr[hg19] 22q11.21(18,648,855-21,461,017)x3 | 2.8Mb | 22q11.2 duplication syndrome | N/S | P/LP | Live birth |
| 7 | 3.70 | Nomal | Nomal | arr[hg19] 12q11q13.12(38,190,102-49,316,412)×2 hmz； 12q23.3q24.13(105,077,945-113,923,939)×2 hmz | 11.1Mb、8.8Mb | AOH | N/S | Others-AOH | Live birth |
| 8 | 3.00 | Nomal | Nomal | arr[hg19] 1q25.1q25.2(175,146,449-176,164,670)x3；Xq26.2(131,028,807-131,820,272)x1 | 1.0Mb、791kb | 3OMIM genes；4 OMIM genes (eg.FRMD7) | N/S | VOUS/P | Live birth |
| 9 | 4.10 | Nomal | Nomal | arr[hg19] 2q14.2q21.2(121,804,081-133,300,702)×2 hmz | 11.5Mb | AOH | N/S | Others-AOH | Live birth |
| 10 | 2.90 | Nomal | Nomal | arr[hg19] 8p22(15,868,963-17,452,339)x3 | 1.6Mb | 8 OMIM genes | N/S | VOUS | Live birth |
| 11 | 3.60 | Nomal | Nomal | arr[hg19] 8q12.1(59,700,319-61,484,612)x3 | 1.8Mb | 3 OMIM genes | N/S | VOUS | Live birth |
| 12 | 3.70 | Nomal | Nomal | arr[GRCh37] 14q22.1q23.3(53159576_65724107)x2 hmz | 12.6Mb | ROH | N/S | Others-ROH | Live birth |
| 13 | 3.30 | Nomal | Nomal | arr[hg19] 10q26.11q26.12(120,890,519-122,179,122)x3 | 1.3Mb | 8 OMIM genes (eg.BAG3) | N/S | VOUS | Live birth |
| 14 | 2.40 | Nomal | Nomal | arr[hg19] 4q12q13.1(58,193,591-62,730,657)x3 | 4.5Mb | ADGRL3 | N/S | VOUS | Live birth |
| 15 | 3.10 | Nomal | Nomal | arr[hg19] 4p16.3(68,345-2,181,924)x1 | 2.1Mb | Wolf-Hirschhorn syndrome | N/S | P | TOP |
| 16 | 3.20 | Nomal | Nomal | arr[hg19] 3q11.1q11.2(93,519,464-96,949,316)x1 | 3.43Mb | 5 OMIM genes | N/S | VOUS | Live birth |
| 17 | 3.20 | Nomal | Nomal | arr[hg19] 22q11.21(18,648,855-21,800,471)x1 | 3.2Mb | DiGeorge syndrome | N/S | P | TOP |
| 18 | 2.90 | Nomal | Nomal | arr[hg19] 10q11.22q11.23(48,750,425-52,457,367)x3；20p12.1(13,963,727-17,657,339)x1 | 2.6Mb、3.7Mb | 17 OMIM genes；9 OMIM genes | maternal | VOUS/VOUS | Live birth |
| 19 | 2.80 | Nomal | Nomal | arr[hg19] 14q22.1q23.3(51,437,619-66,297,540)×2 hmz | 14.9Mb | AOH | De novo | Others-AOH | Live birth |
| 20 | 4.80 | Nomal | Nomal | arr[hg19] 16p13.11(14,910,158-16,458,424)x1 | 1.5Mb | 16p13.11 microdeletion syndrome | N/S | P | TOP |
| 21 | 2.60 | Nomal | Nomal | arr[hg19] 16p13.11p12.3(15,338,152-18,172,468)x3 ma | 2.9Mb | 16p13.11 duplication syndrome | maternal | VOUS | Live birth |
| 22 | 2.70 | Nomal | Nomal | arr[hg19] 1q21.1q21.2(145,895,746-147,830,830)x1 | 1.9Mb | 1q21.1 microdeletion syndrome | maternal? | P | Live birth |
| 23 | 3.60 | Nomal | Nomal | arr[hg19] Xp11.4(40,018,086-40,946,284)x2 | 928kb | 4 OMIM genes | maternal? | VOUS | Live birth |
| 24 | 2.70 | Nomal | Nomal | arr[hg19] Xq22.3(104,675,923-105,815,760)x1 | 1.1Mb | 3 OMIM genes | N/S | VOUS | Live birth |
| 25 | 2.70 | Nomal | Nomal | arr[hg19] 21q11.2q21.1(15,961,155-17,519,310)x1 | 1.6Mb | 3 OMIM genes | N/S | VOUS | Live birth |
| 26 | 3.10 | Nomal | Nomal | Maternal UPD (7) mosaics | - | Russell-Silver syndrome | maternal | Others-mosaics | Live birth |
| 27 | 9.00 | Nomal | Nomal | arr(1-22)×2,(X)×1 | - | Turner syndrome | N/S | P | Live birth |
| 28 | 3.10 | Nomal | Nomal | arr[GRCh37] 1q21.1q21.2(146586250_147391923)x3 | 806kb | 1q21.1 microdeletion syndrome | De novo | P | Live birth |
| 29 | 2.70 | Nomal | Nomal | arr[GRCh37] 12p13.33p13.32(2024105_3841392)x1 | 1.8Mb | Timothy syndrome（CACNA1C） | N/S | VOUS | Live birth |
| 30 | 4.00 | Nomal | Nomal | arr[GRCh37] 14q32.31q32.33(101858740_107279475)x2 hmz | 5.4Mb | ROH | De novo | Others-ROH | Live birth |
| 31 | 2.50 | Nomal | Nomal | arr[GRCh37] 16p11.2(29591327_30167919)x3 | 577kb | 16p11.2 microduplication syndrome | N/S | P | Live birth |
| 32 | 3.00 | Nomal | Nomal | arr[GRCh37] 16p11.2(29428532_30190029)x1 | 761kb | 16p11.2 microdeletion syndrome | N/S | P | TOP |
| 33 | 3.11 | Nomal | Nomal | arr[GRCh37] 16p13.11(15058821_16309046)x3 | 1.25Mb | 10 OMIM genes | maternal? | VOUS | Live birth |
| 34 | 2.90 | Nomal | Nomal | arr[GRCh37] 16p11.2(29581102_30190029)x1 | 609kb | 16p11.2 microdeletion syndrome | N/S | P | Live birth |
| 35 | 3.00 | Nomal | Nomal | arr[GRCh37] 16p12.2(21841354_22431031)x1 | 590kb | 16p12.2 microdeletion syndrome | maternal | P | Live birth |
| 36 | 3.60 | Nomal | Nomal | arr[GRCh37] 14q23.2q23.3(64289920_66098274)x3 | 1.8Mb | 14 OMIM genes | N/S | VOUS | Live birth |
| 37 | 3.50 | Nomal | Nomal | arr[GRCh37] 16p12.2(21946956_22442007)x3 | 495kb | 4 OMIM genes | N/S | VOUS | Live birth |
| 38 | 3.10 | Nomal | Nomal | arr[hg19] 22q11.21(18,648,855-20,312,661)x3 | 1.7Mb | 22q11.2 duplication syndrome | N/S | P | TOP |
| 39 | 2.80 | Nomal | Nomal | arr[hg19] 16p13.11(15,171,146-16,272,403)x3 | 1.1Mb | 16p13.11 microduplication syndrome | N/S | VOUS | Live birth |
| 40 | 3.20 | Nomal | Nomal | arr[hg19] 22q11.21(18,648,855-21,454,872)x3 | 2.8Mb | 22q11.2 microduplication syndrome | N/S | P | Live birth |
| 41 | 3.00 | Nomal | Nomal | arr(22)×2-3 | - | Mosaics 22 Trisomy（74%） | N/S | Others-mos | TOP |
| 42 | 4.40 | Nomal | Nomal | arr[hg19] 16p13.11(14,892,976-16,538,596)x1 | 1.65Mb | 16p13.11 microdeletion syndrome | N/S | P | Live birth |
| 43 | 3.00 | Nomal | Nomal | arr[hg19] 13q12.12(23,519,917-24,922,373)x3 | 1.4Mb | 8 OMIM genes | N/S | VOUS | Live birth |
| 44 | 2.60 | Nomal | Nomal | arr[hg19] 2q12.3q13(109,373,186-110,442,979)x3 | 1.07Mb | 3 OMIM genes | N/S | VOUS | Live birth |

mm, millimeter; NT, nuchal translucency; CMA, chromosomal microarray analysis; N/S, not specified;P, Pathogenic; VOUS, variant of uncertain significance;TOP, termination of pregnancy ; UPD,uniparental disomy.

Table S2. Clinical and molecular information of 40 fetuses with increased NT identified by WES

| **Case ID** | **NT（mm)** | **QF-PCR result** | **CMA result** | **WES result** | **Zygosity** | **Inheritance** | **Disease association(s)** | **Categorization** | **outcome** |
| --- | --- | --- | --- | --- | --- | --- | --- | --- | --- |
| 1 | 3.10 | Nomal | Nomal | PTPN11(NM_002834)c.1124A>G(p.Y375C) | Heterozygous | maternal  AD | Noonan syndrome 1/LEOPARD syndrome 1/Metachondromatosis | VOUS | Live birth |
| 2 | 3.40 | Nomal | Nomal | CACNA1E(NM_000721)c.4615C>T(p.R1539*)；PITX1(NM_002653)c.297_298delinsTT(p.Q99_Q100delinsH*) | Heterozygous | De novo  AD | Developmental and epileptic encephalopathy 69 / Liebenberg syndrome | LP/LP | Live birth |
| 3 | 4.50 | Nomal | Nomal | PEX1(NM_000466)c.1583_1587delTACAA(p.I528Sfs*13)；PEX1(NM_000466)c.782_783delAA(p.Q261Rfs*8) | Compound Heterozygous | Paternal/Maternal  AR | Peroxisome biogenesis disorder 1A/1B | LP/VOUS | Live birth |
| 4 | 4.40 | Nomal | Nomal | LZTR1(NM_006767)c.2306C>T(p.T769M) | Heterozygous | Maternal  AD | Noonan syndrome 10 | VOUS | Live birth |
| 5 | 3.20 | Nomal | Nomal | FGFR3(NM_000142)c.1138G>A(p.G380R) | Heterozygous | De novo  AD | Achondroplasia | P | Live birth |
| 6 | 3.60 | Nomal | Nomal | XYLT1(NM_022166)c.2456G>T(p.G819V) | Homozygous | Paternal/Maternal  AR | Desbuquois dysplasia 2 | VOUS | TOP |
| 7 | 3.20 | Nomal | Nomal | SOS1(NM_005633)c.929G>A(p.R310H) | Heterozygous | Maternal  AD | Noonan syndrome 4 | VOUS | Live birth |
| 8 | 3.10 | Nomal | Nomal | PRRX1(NM_022716)c.146T>C(p.V49A) | Heterozygous | De novo  AD/AR | Agnathia-otocephaly complex | VOUS | Live birth |
| 9 | 3.40 | Nomal | Nomal | RAI1(NM_030665)c.3445C>T(p.Arg1149Cys) | Heterozygous | De novo  AD | Smith-Magenis syndrome | VOUS | Live birth |
| 10 | 5.60 | Nomal | Nomal | COL2A1(NM_001844)c.3599G>T(p.Gly1200Val) | Heterozygous | De novo  AD | Achondrogenesis, type II or hypochondrogenesis | VOUS | TOP |
| 11 | 3.40 | Nomal | Nomal | ETFDH(NM_004453.4)c.920C>G(p.Ser307Cys)；ETFDH(NM_004453.4)c.959C>T(p.Ala320Val) | Compound Heterozygous | Paternal/Maternal  AR | Multiple Acyl-CoA Dehydrogenase Deficiency (MADD) | LP/VOUS | Live birth |
| 12 | 2.70 | Nomal | Nomal | LZTR1(NM_006767.4)c.2263C>T(p.Arg755Trp) | Heterozygous | Maternal  AD/AR | Noonan syndrome 10/Noonan syndrome 2 | VOUS | Live birth |
| 13 | 3.00 | Nomal | Nomal | ZMIZ1(NM_020338.4)c.1342C>T(p.Gln448*) | Heterozygous | Maternal  AD | Neurodevelopmental disorder with dysmorphic facies and distal skeletal anomalies | LP | Live birth |
| 14 | 4.10 | Nomal | Nomal | LZTR1(NM_006767)c.740G>T(p.S247I) | Heterozygous | De novo  AD | Noonan syndrome 10 | LP | TOP |
| 15 | 3.24 | Nomal | Nomal | LZTR1(NM_006767)c.1201T>G(p.Y401D) | Heterozygous | Paternal  AD/AR | Noonan syndrome 10/Noonan syndrome 2 | VOUS | Live birth |
| 16 | 4.10 | Nomal | Nomal | CHD4(NM_001273)c.4018C>T(p.R1340C) | Heterozygous | De novo  AD | Sifrim-Hitz-Weiss syndrome | LP | Live birth |
| 17 | 3.90 | Nomal | Nomal | VARS1(NM_006295)c.94G>T(p.Gly32*)；VARS1(NM_006295)c939G>C(p.Trp313Cys) | Compound Heterozygous | Maternal/PaternalAR | Neurodevelopmental disorder with microcephaly, seizures, and cortical atrophy | LP/VOUS | Live birth |
| 18 | 3.40 | Nomal | Nomal | PACS1(NM_018026) heterozygous deletion of exons 12-24 | Heterozygous | Paternal  AD | Schuurs-Hoeijmakers syndrome | LP | Live birth |
| 19 | 3.60 | Nomal | Nomal | MYH3(NM_002470)c.3402_3403delG(p.K1135Tfs*65) | Heterozygous | De novo  AR/AD | Arthrogryposis, distal, type 2A/type 2B3;Contractures, pterygia, and spondylocarpostarsal fusion syndrome 1A/1B | LP | Live birth |
| 20 | 4.00 | Nomal | Nomal | PTPN11(NM_002834)c.155C>T(p.T52I) | Heterozygous | De novo  AR/AD | Noonan syndrome 1 | LP | Live birth |
| 21 | 3.20 | Nomal | Nomal | TRIP11(NM_004239)c.2138C>A(p.T713N); c.3705G>C(p.M1235I) | Compound Heterozygous | Paternal/Maternal  AR | Achondrogenesis, type IA;Odontochondrodysplasia 1 | VOUS | Live birth |
| 22 | 12.90 | Nomal | Nomal | PHGDH(NM_006623)c.1015C>G(p.L339V),  PHGDH(NM_006623)c.1037G>A(p.W346*) | Compound Heterozygous | Paternal/Maternal  AR | Neu-Laxova syndrome 1;Phosphoglycerate dehydrogenase deficiency | LP/VOUS | TOP |
| 23 | 3.60 | Nomal | Nomal | SOS1(NM_005633)c.508A>G(p.K170E) | Heterozygous | De novo  AD | Noonan syndrome 4 | P | TOP |
| 24 | 3.90 | Nomal | Nomal | SOS1(NM_005633)c.587C>T(p.S196L);  COL3A1(NM_000090)c.1682G>A(p.G561D) | Heterozygous | Paternal/De novo  AD/AD | Noonan syndrome 4;Ehlers-Danlos syndrome, vascular type | VOUS/VOUS | Live birth |
| 25 | 3.17 | Nomal | Nomal | LZTR1(NM_006767)c.2075T>C(p.F692S) | Heterozygous | Maternal  AD/AR | Noonan syndrome 10/Noonan syndrome 2 | VOUS | Live birth |
| 26 | 4.10 | Nomal | Nomal | STS(NM_000351.6)c.463G>A(p.G155S) | Hemizygous | Maternal  XLR | Ichthyosis, X-linked | VOUS | Live birth |
| 27 | 6.00 | Nomal | Nomal | LZTR1(NM_006767)c.2325+1G>A | Heterozygous | Paternal  AD/AR | Noonan syndrome 10/Noonan syndrome 2 | VOUS | Live birth |
| 28 | 5.70 | Nomal | Nomal | NSD1(NM_022455)c.1525_1540del(p.Thr509Ala fs*22) | Heterozygous | De novo  AD | Sotos syndrome 1 | P | Live birth |
| 29 | 6.50 | Nomal | Nomal | FGFR3(NM_001163213)c.742C>T(p.Arg248Cys) | Heterozygous | De novo  AD | Achondroplasia/Thanatophoric dysplasia, type I | P | Live birth |
| 30 | 3.72 | Nomal | Nomal | TCIRG1(NM_006019)c.1037_1040dupGTGC(p.Val348Cysfs*143),(NM_006019)c.2218_2219delCT(p.Leu740Glufs*90) | Compound Heterozygous | Paternal/Maternal  AR | Osteopetrosis, autosomal recessive 1 | VOUS | Live birth |
| 31 | 4.40 | Nomal | Nomal | SOS2(NM_006939.3)c.20C>G(p.Pro7Arg) | Heterozygous | Paternal  AD/AR | Noonan syndrome 9 | VOUS | Live birth |
| 32 | 4.10 | Nomal | Nomal | HRAS(NM_005343.3)c.351G>T(p.Lys117Asn) | Heterozygous | De novo  AD | Costello syndrome | VOUS | TOP |
| 33 | 11.80 | Nomal | Nomal | SOX9(NM_000346.4)c.788delG(p.Gly263Alafs*16) | Heterozygous | De novo  AD | Campomelic Dysplasia | LP | Live birth |
| 34 | 5.10 | Nomal | Nomal | COL2A1(NM_001844.5)c.4196del(p.Tyr1399Phefs*36) | Heterozygous | De novo  AD | Achondrogenesis, type II | LP | TOP |
| 35 | 3.20 | Nomal | Nomal | GRIN2B(NM_000834.4)c.655C>T(p.Gln219*) | Heterozygous | De novo  AD | Developmental and epileptic encephalopathy 27;Intellectual developmental disorder, autosomal dominant 6, with or without seizures | LP | Live birth |
| 36 | 4.20 | Nomal | Nomal | EPHB4(NM_004444.5)c.805C>T(p.Arg269*) | Heterozygous | Paternal  AD | Capillary malformation-arteriovenous malformation 2;Lymphatic malformation 7 | VOUS | Live birth |
| 37 | 3.00 | Nomal | Nomal | FGFR3(NM_000142.4)c.742C>T(p.Arg248Cys) | Heterozygous | De novo  AD | Achondroplasia/Thanatophoric dysplasia, type I | P | Live birth |
| 38 | 5.00 | Nomal | Nomal | PTPN11(NM_002834.4)c.124A>G(p.Thr42Ala) | Heterozygous | De novo  AD | Noonan syndrome 1/LEOPARD syndrome 1/Metachondromatosis | P | TOP |
| 39 | 3.10 | Nomal | Nomal | LZTR1(NM_006767.4)c.27delG(p.Gln10Argfs*15) | Heterozygous | Paternal  AD | Noonan syndrome 10/Noonan syndrome 2/Schwannomatosis-2 | P | Live birth |
| 40 | 5.00 | Nomal | Nomal | MAP2K1(NM_002755.4)c.608A>G(p.Glu203Gly) | Heterozygous | De novo  AD | Cardiofaciocutaneous syndrome 3 | P | Live birth |

mm, millimeter; NT, nuchal translucency; TOP, termination of pregnancy ; QF-PCR,quantitative fluorescent polymerase chain reaction;WES, Whole Exome Sequencing; CMA, chromosomal microarray analysis; P, Pathogenic;LP,Likely Pathogenic; VOUS, variant of uncertain significance;AD, autosomal dominant; AR, autosomal recessive.
